# Penile Glans Amputation following Circumcision: A Case Report of a Rare Complication

**DOI:** 10.1155/2020/5806987

**Published:** 2020-10-02

**Authors:** Salman Soltani, Mahdi Mottaghi, Amir Jafarpisheh, Mahmoud Tavakkoli

**Affiliations:** ^1^Kidney Transplantation Complications Research Center, Mashhad University of Medical Sciences, Mashhad, Iran; ^2^Students Research Committee, Faculty of Medicine, Mashhad University of Medical Sciences, Mashhad, Iran; ^3^Department of Urology, Faculty of Medicine, Mashhad University of Medical Sciences, Mashhad, Iran; ^4^Kidney Transplantation Complications Research Center, Mashhad University of Medical Sciences, Mashhad, Iran

## Abstract

Circumcision is the most prevalent surgery among men. Like any other surgical intervention, it is associated with several complications. A rare shocking complication is glans amputation which is a urologic emergency. Herein, we present a 4-year-old boy with penile glans amputation following circumcision. The reimplantation was performed in less than two hours. We approximated the ends over a size 4 : 0 catheter. The urethral anastomosis was performed via 6 : 0 Vicryl sutures. Then, we sutured amputated glans in place via Vicryl 4 : 0. We immobilized the penis for a week via bandages used in penile reconstruction surgeries. We also used pentoxifylline to treat glans ischemia after surgery. The patient and his parents did not mention any difficulties or abnormalities while voiding, and the cosmetic result was favorable after three months of follow-up.

## 1. Introduction

Circumcision is an ancient surgical procedure and remains the most frequent surgery until today. About 25 to 35% of the male population around the world are circumcised [1–3]. Muslims, Jews, Christians (especially in North America), and native Australians are accounted for the majority of this fraction [4].

## 2. Case Presentation

**Figure 1:**
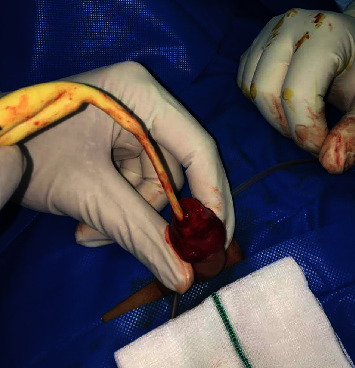
Amputated glans and complete injury of the urethra.

A 4-year-old boy was brought to the emergency department due to the glans amputation during the circumcision procedure. The procedure performed by a general practitioner in an outpatient office, using the Mogen clamp. The responsible physician had covered the amputated part in sterile moist gauze and a plastic bag, then put it in an ice pack and urgently sent him to our tertiary referral hospital where he was prepared for surgery in less than an hour (Figure 1).

**Figure 2:**
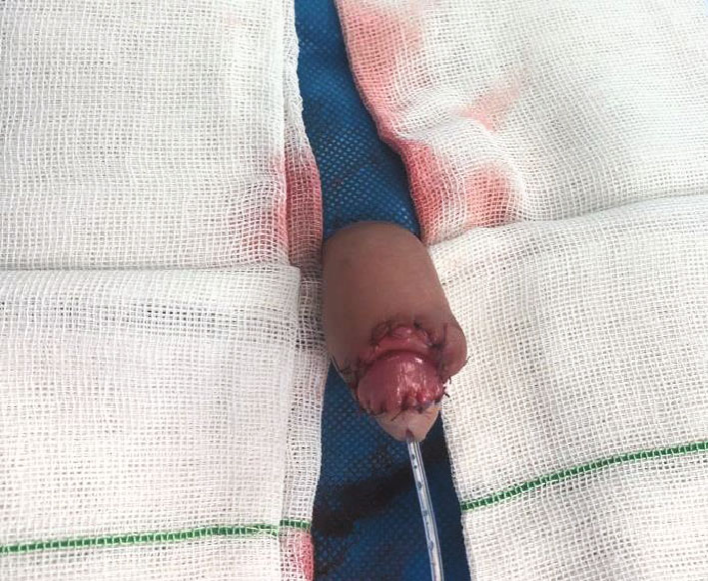
Penis pink appearance in the postoperation room.

**Figure 3:**
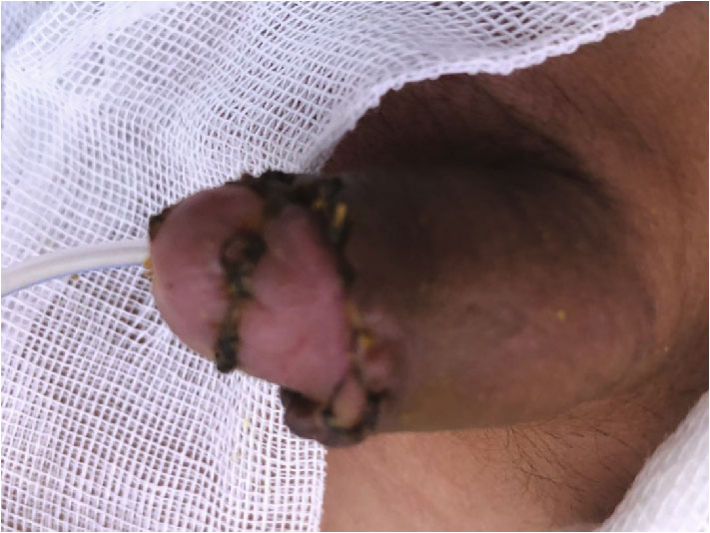
Follow-up image after 3 weeks of operation.

In the operating room, both amputated surfaces were irrigated via 0.9% saline and 10% povidone-iodine. The amputated part appeared completely white. After the preparation for replantation, a 6 Fr transurethral catheter inserted through the amputated part of the glans and then inserted further to the urethra and bladder. Urethra anastomosed via interrupted sutures (6 : 0 Vicryl suture). Then, the amputated part of the glans reattached to the penis via Vicryl sutures 4 : 0 and finally, circumcision completed. After an hour in the postoperation room, the first signs of reperfusion started to appear like bright pink appearance, but poor capillary reperfusion (about 2-3 seconds) (Figure 2). The patient's vital signs were stable and he was transferred to the ward. The penis immobilized via a splint. The next morning, parents complained about minor discoloration of the tip of the glans. After consultation with a vascular surgeon, we started him on pentoxifylline (200 mg twice a day, orally). We also managed the wound by keep soaking the bandages and the gauzes. We carefully watched the glans penis appearance, and after two days, the discoloration faded. The patient was discharged home one week after the surgery with a Foley catheter and antibiotics. After 21 days, the Foley catheter was removed while the appearance of the penis was normal. Ultrasound showed normal urinary residue. We also performed a urinary flowmetry which was normal. After three months, his parents reported normal voiding stream and examination revealed no stenosis with satisfactory cosmetic results. Parents look agitated during all visits and did not consent for taking a follow-up image and said that it makes their son uncomfortable. Finally, they agreed to take a picture themselves (Figure 3).

## 3. Discussion

Although several medical benefits are proposed for circumcision, it is mainly done due to religious beliefs or as a routine cultural behavior [4]. Medically, it decreases urinary tract infection (especially in the first year of life), penile cancer, cervical cancer in one's partner, and sexually transmitted infections (HSV, HPV, HIV, trichomoniasis, syphilis, and chancroid) [5]. The Joint United Nations Programme on HIV and AIDS (UNAIDS) advises performing circumcision to prevent HIV. It provides lifetime partial protection against HIV transmission from female to male. It is advised to be performed in the first month of life because of its less complication rate during this period [5]. Circumcision's complication rate was about 0.19% to 3.1% which reported in a systematic review in 2010 [6]. Interventions of the health care system on training the circumcisers probably affect the complication rate. In Israel where traditional circumcisers (Mohels) are trained by the ministry of health and ministry of religion, the complication rate is about 0.34%, while in Nigeria, this rate is about 20% and this high complication rate is probably due to inadequate training of circumcisers [2, 4]. Circumcision's complications might present early or delayed. Bleeding and infection are the most frequent early complications which are generally easy to control [2, 4]. Delayed complications include excessive foreskin, shortage of penile skin, skin bridges, fistula, buried penis, meatal stenosis, and glans injures [2, 4]. Penile glans amputation is rare but it is a urologic emergency and needs prompt replantation. The surgeon should focus on restoring the function of the glans and urethra while preserving the cosmetic appearance of the glans to avoid the future psychological burden. The amputated part should be dressed via a sterile moist gauze in a plastic bag and kept in the ice and water. Direct contact of the amputated part with the ice can cause tissue damage and replantation failure [1, 7]. The replantation should be performed under eight hours from injury to achieve the best functional and cosmetic results [1, 8]. A urinary catheter should be placed for at least a week to decrease the tension on the urethra and subsequently decrease the chance of fistula development [7].

The etiology of glans amputation during circumcision is not well understood but Salle et al. found a similar pattern of penis injury (the ventrolateral aspect of the glans is cut accompanying urethral injury) and remnant adhesions in one amputated glans [1]. They hypothesized that remaining adhesions between prepuce and glans, especially on the ventral aspect of the penis, can cause clamping the glans partially or completely. They also declare that poor releasing of adhesions on the ventral side might be due to the location of the frenular artery. Injury to this artery can cause profuse bleeding; therefore, inexperienced circumcisers might avoid this area to reduce the probability of bleeding; thus, more adhesions remain [1]. Another risk factor for glans amputation is the technique in which the circumcision performed. There are three basic patterns for circumcision: Guillotine-like clamps (like Mogen clamp), glans protective clamps (like Plastibell and Gomco clamps), and free-hand approach (usually used in the operating room) with no device. Each type is associated with advantages and disadvantages. Mogen clamp is commonly used because it is time-saving and provides good cosmetic outcome. But its major disadvantage is that glans is hidden during clamping and cutting the prepuce [1]. The experience of the circumciser is another determining factor for the complication rate. Circumcision is usually performed by traditional circumcisers, nurses, general practitioners, and rarely surgeons and urologists [2]. The study of Okeke et al. showed that traditional circumcisers had lower complication rate but it is probably due to underestimation. Traditional circumcisers probably report only major complications [2].

For treatment, it is advised to perform penile shaft replantation in a microvascular fashion, but glans vessels are low-gauge and macroscopic replantation seems to be the best choice [8, 9]. Urethral injury almost always a company glans amputation. Reanastomosis of the urethra decreases the chance of fistula and meatal stenosis but increases the chances of urethral shortage and subsequent ventral chordee [9]. Faydaci et al. performed replantation without urethral reanastomosis to gain better cosmetic outcomes [9].

Glans discoloration and edema were seen in the same studies on the second postoperative day [7, 9]. Different strategies were used in different settings. Faydaci et al. used a hyperbaric oxygen chamber; Roche et al. used leech medicinal therapy; and we used pentoxifylline after consultation with vascular surgeons, and all methods provide satisfactory outcomes [7, 9]. As expected, patients' parents act agitatedly during the treatment course and a lack of a psychologist to control the situation caused several difficulties for the patient, his parents, and physicians. Glans amputation can cause psychological complications (short and long term) in the patient and his family, so we suggest referring the patients to get further consultation and support from a psychologist.

## 4. Conclusion

Penile glans amputation is a rare complication of circumcision. It can be prevented by educating circumcisers. Guillotine-like procedures increase the risk and proper removal of adhesions, especially on the ventral aspect of the penis might decrease the risk. Treatment is replantation of amputated part within eight hours of the accident. Hyperbaric oxygen chamber, medicinal leech therapy, and pentoxifylline can be used to provide reperfusion to the amputated part after the replantation.
